# Emotional impact in β-thalassaemia major children following cognitive-behavioural family therapy and quality of life of caregiving mothers

**DOI:** 10.1186/1745-0179-5-5

**Published:** 2009-02-23

**Authors:** Luigi Mazzone, Laura Battaglia, Francesca Andreozzi, Maria Antonietta Romeo, Domenico Mazzone

**Affiliations:** 1 Division of Child Neurology and Psychiatry, Department of Pediatrics, University of Catania, Catania, Italy

## Abstract

**Background:**

Cognitive-Behavioural Family Therapy (CBFT) can be an effective psychological approach for children with β-thalassaemia major, increasing compliance to treatment, lessening the emotional burden of disease, and improving the quality of life of caregivers.

**Design and methods:**

Twenty-eight β-thalassaemic major children that followed CBFT for one year were compared with twenty-eight age-matched healthy children, focusing particularly on behavioural, mood, and temperamental characteristics as well as compliance with chelation, assessed using the Child Behaviour Checklist (CBCL), Children's Depression Inventory (CDI), Multidimensional Anxiety Scale for Children (MASC), and Emotionality, Activity, Sociability and Shyness Scale (EAS). We also monitored the quality of life of caregiving mothers using the World Health Organization Quality Of Life (WHOQOL-BREF) questionnaire. Data were analysed with non-parametric standard descriptive statistics.

**Results:**

90% of β-Thalassaemic children showed good compliance with chelation therapy; however they had significantly increased somatic complains, physical symptoms and separation panic. Moreover, temperamental assessment revealed high emotionality and poor sociability in treated thalassaemic children and in their mothers. Physical and psychological domains concerning individual's overall perception of quality of life resulted impaired in mothers of β-thalassaemic children.

**Conclusion:**

CBFT can be a valid tool to increase the compliance with chelation therapy in β-thalassaemic children; however, treated children continue to show an important emotional burden; moreover, CBFT therapy seems not to have any positive impact on the quality of life of caregiving mothers, who may therefore need additional psychological support.

## Background

β-Thalassemia major is a disorder characterized by defective production of hemoglobin and excessive destruction of red blood cells. Hemoglobin (Hb) is formed of four protein subunits, two α and two β. Genetic mutations in the gene encoding for the β subunits of the protein, result in reduced or totally absent synthesis of the globin β-chains, leading to the formation of abnormal hemoglobin or even to the absence of β hemoglobin. This defect causes an abnormal development of red blood cells and ultimately anemia, which is the characteristic symptom of the thalassemia. The disease is prevalent among Mediterranean people; the highest frequency is found in the Greek islands, in Italy (lower Pò valley, Sicily and Sardinia) and in Asia, where the highest concentration of people carrying the genetic mutations underlying thalassemia is found in the Maldives.

The most severe form is the β-thalassemia major, which is characterized by a severe microcytic, hypochromic anemia (Cooley's anemia), whose symptoms appear usually within the first 2 years of life. Infants become pale and asthenic, have a poor appetite, grow slowly, and often develop jaundice; spleen, liver, and heart may also be enlarged. Adolescents with the most severe form may experience delayed puberty. The usual treatment consists of periodic blood transfusions, that can cause iron overload within tissues. Children on hypertransfusion regimes will maintain normal growth up to puberty. Serum ferritin gives an estimate of the total body iron; levels higher than 2500 mg/l over a period of 15 years are considered a risk factor for cardiac disease.

Desferrioxamine is the most widely used iron chelator and it is shown to reduce hepatic iron and to improve hepatic fibrosis and cardiac dysfunction. The toxic effects of desferrioxamine are well documented. If iron chelation is inadequate, deposition can occur in several endocrine organs, thus leading to disease conditions such as diabetes mellitus and hypogonadotrophic hypogonadism and growth hormone deficiency, and less often, to hypothyroidism and hypoadrenalism. Bone marrow transplantation is a radical care option for β-thalassemia major, but it can induce some complications, including chronic graft versus host disease, mixed chimaerism, hepatic iron overload, cardiac disease and viral hepatitis [1,2].

In children, especially, β-thalassemia major and its complications carry a significant psychological impact, causing emotional burden, hopelessness, and difficulty with social integration. Thalassemic children have been described to show impaired abstract reasoning, deficits of language, attention, memory, constructional/visual spatial skills, and executive functions, all of which are more prominent in hemosiderotic subjects [3]. In most children, low intelligence quotient appears to be correlated with poor school performances and physical or social restrictions for the severity and chronicity of the disease, and not with age, sex, ferritin level, brainstem auditory, visual and somatosensory evoked potentials, or motor and sensory nerve conduction velocity [4,5].

Thalassemic children feel different from their peers and elaborate negative thoughts about their life, guilt senses, increased anxiety and low self-esteem; their behavioural profile is similar to normal subjects, but many of them can manifest severe psychosocial problems due to difficulties in complying with the painful chelation; male patients, in particular, show oppositional defiant disorder [6-9]. Within the family, concerns for the future of the thalassemic child may contribute to worsen the relationship between members, and to increase marginalization and isolation. A psychological support seems therefore to be useful to reduce the emotional burden of β-thalassemia major children and their families. Additionally, quality of life, which is an index of health care defined as an individual's perception of their position in life in the context of culture and value systems in which they live, and in relation to their goals, expectations, standards, and concerns, is often limited by the chronic illness.

Even if in recent years the innovative therapeutic strategies for thalassaemia major have greatly increased the life expectancy, the fear for the disease itself and for the possible complications of invasive medical procedures still affect the quality of life of the entire family. So far, only a few studies have been conducted analyzing the quality of life in β-thalassaemia, and these studies have employed two instruments for the assessment of this parameter: one derived from the WHOQOL-100 questionnaire, and another one designed specifically for thalassemia to assesses both psychosocial and clinical burden [10,11].

In our study we have structured a psychological support for β-thalassaemia major children using Cognitive-behavioural Family Therapy (CBFT), in order to facilitate the adherence to iron chelation and to contain the emotional burden. We have included caregiving mothers in CBFT sessions as maternal participation could be a useful strategy to improve mother/child communication, and ultimately to promote a greater compliance to chelation therapy and to improve the quality of their life [12]. To prevent the development of clinically manifest psychosocial morbidity and to improve the quality of life of the family are, consequently, important goals in the treatment of β-thalassaemia [13].

The objectives of the present study were the following:

1. To evaluate the compliance of the chelation treatment in β-thalassaemia major children after a sessions programme of CBFT;

2. To assess behaviour, mood, and temperament peculiarities of patients, compared to healthy subjects;

3. To analyze quality of life (QoL) of caregiving mothers.

## Methods

### Study design

Twenty-eight outpatients with a diagnosis of β-thalassaemia major (aged 12.79 ± 3.57 years; 15 subjects > 13 years) were included in the study. Diagnosis of β-thalassaemia major was made in each patient before the age of 3 years old. These patients received an high haemo-transfusional regimen and a chelation therapy with desferioxamine, which was infused subcutaneously 10–12 hours a day for 5–6 days a week (25–50 mg/kg). Twenty-eight healthy subjects (aged 12.52 ± 3.48 years;14 subjects > 13 years) were also enrolled from a database of children attending a well-being paediatric clinic for routine checks. Both thalassemic and healthy groups of children received a comprehensive physical and neurological assessment, including EEG and ophthalmological examinations. β-thalassaemia major children were submitted to CBFT for 1 year with their mothers. CBFT was carried out according to Dattilo and Montano [14], and CBFT sessions were held by a psychologist twice a week for each month, often overlapping with an hemo-transfusion or a laboratory check-up. Each CBFT session lasted 45 minutes, with a global duration of 900 min corresponding to 15 hours of therapy for each pair. There was no attrition.

Specific targets of CBFT were:

1. to communicate informations on the biological aspects and on the emotional impact of the disease; to increase the knowledge of possible side-effects of the chelation treatment as well as of myths and misconceptions about the treatment itself;

2. to identify relevant psychosocial factors;

3. to manage and to minimize emotional suffering of thalassemic children;

4. to improve mother/child communication to foster a positive relationship;

5. to help patient and mother to cope with events occurring in the family, and to realize that erroneous beliefs could cause emotional distress.

After 1 year of CBFT, β-thalassaemia major children were evaluated for compliance index (as a percentage), calculated as the ratio between days of administration of therapy and days of prescription within the last 1 year; an index higher than 75% indicated a good compliance. All participants were also submitted to a battery of the following psychological instruments for a comprehensive evaluation of behaviour, mood, and temperament compared to healthy subjects:

a) Wechsler Intelligence Scale for Children (WISC-IV) for the intelligence quotient (IQ) [15].

b) Child Behaviour Checklist (CBCL), completed by the parents rating behavioural and emotional problems along two dimensions of 'internalising' symptoms such as anxiety and depression, and 'externalising' symptoms, such as aggression and hyperactivity. Raw scores on each clinical factor were transformed to T-scores based on published norms [16].

c) Multidimensional Anxiety Scale for Children (MASC), completed by the child to score symptoms of anxiety according to a 4-point Likert-style self-report scale. Sub-factors include physical symptoms, harm avoidance, social anxiety, and separation anxiety. Raw scores were converted into standard T-scores [17].

d) Children's Depression Inventory (CDI), completed by the child to rate symptoms of depression. The CDI is a self-rating scale scored on three-point scale (0 absent; 1 moderate; 2 severe) reflecting severity of symptoms. Total score ranges from 0 to 54; 19-point cut-off indicates the threshold discriminating children at risk of depression [18].

e) Emotionality Activity Sociability and Shyness (EAS) Scale was administered to children and their caregiving mothers to rate four temperamental traits: emotionality, activity, sociability, and shyness. For each item the subject was asked to rate the statement on a "5 point" Likert scale, ranging from 0 (not characteristic or typical) to 5 (very characteristic or typical) [19].

f) World Health Organization Quality Of Life (WHOQOL)-Bref questionnaire was administered to mothers of β-thalassaemia major children and mothers of normal subjects [11]. It has 26 items and four domains related to quality of life: physical health, psychological health, social relationships and environment, and two individual items covering overall quality of life and general health. Higher scores denote better QoL. The WHOQOL-Bref domain scores showed good discriminant validity and internal consistency of subscales evaluated by Cronbach's alpha coefficients (alpha P ≥ 0.75). Data were analysed with standard descriptive statistics: χ^2 ^test and non-parametric instrument Student's t-test. A two-tail *p*-value of < .05 was used as the cut-off point of statistical significance. Informed consent was obtained from children and their mothers.

## Results

Twenty-eight β-thalassaemic children submitted to CBFT were compared to twenty-eight age-matched healthy subjects. The two groups, statistically compared by a χ^2 ^test, were homogeneous for mean age, male/female ratio, socio-economic status, education, and I.Q.. No family history of psychological illness was reported in any subject and none of them showed any physical, neurological, ophtalmological, or EEG alteration.

Compliance to treatment of β-thalassaemic children was good in 25/28 patients (greater than 90%).

Compared to healthy subjects, β-thalassaemic children displayed more somatic complaints as measured on the CBCL (58.29 ± 7.37 vs. 53.32 ± 4.48, p = 0.004) and more physical symptoms (48.79 ± 6.37 vs. 45.04 ± 3.52, p = 0.009) and separation panic (53.36 ± 10.31 vs. 48.18 ± 6.96, p = 0.032) as measured by the MASC. CDI scores were not statistically different between β-thalassaemic children and healthy subjects; however, in 3 patients CDI scores exceeded the cut-off. On the EAS thalassemic patients displayed high emotionality (p = 0.031) and low sociability (p < 0.001); and their mothers displayed high emotionality and shyness, and low sociability (table 1).

**Table 1:** EAS scores (mean ± SD) in β-thalassaemic major children and their mothers and in healthy subjects.

EAS ^for children^	β-Thalassaemic patients (28)	Normal subjects (28)	p-value^a^
Emotionality	2.80 ± 0.91	2.29 ± 0.81	0.031
Activity	3.60 ± 0.74	3.69 ± 0.59	n.s.
Sociability	2.74 ± 0.51	3.50 ± 0.70	0.001
Shyness	2.60 ± 0.75	2.34 ± 0.49	n.s.
			
EAS ^for mothers^			
Emotionality	2.98 ± 0.71	2.40 ± 0.28	0.001
Activity	3.19 ± 0.61	3.25 ± 0.45	n.s.
Sociability	3.45 ± 0.52	4.13 ± 0.59	0.001
Shyness	2.63 ± 0.78	2.10 ± 0.69	0.009

^a ^For statistical comparison student's t-test has been used.

Responses to the WHOQOL-Bref questionnaire indicated that mothers of β-thalassaemic children showed impairment in the following domains: physical health, psychological health, quality of life and general health (table 2).

**Table 2:** WHOQOL-Bref scores (mean ± SD) in mothers with β-thalassaemic major children vs. normal group mothers; Higher scores indicate a higher QOL.

Domains	β-Thalassemia Group Mothers (N = 28)	Normal Group mothers (N = 28)	p-value^a^
Physical Health	57.29 ± 11.47	68.45 ± 15.68	0.004

Psychologic Health	63.80 ± 9.22	69.59 ± 7.43	0.012

Social Relationships	74.01 ± 14.25	79.37 ± 10.01	0.109

Enviroment	48.54 ± 14.41	50.85 ± 1.47	0.402

Quality of Life	62.50 ± 15.96	77.98 ± 13.75	0.001

General Health	58.04 ± 19.31	71.43 ± 21.08	0.016

^a ^For statistical comparison student's t-test has been used.

## Discussion

β-thalassaemia is a chronic illness that causes excessive psychological burden to children and their families as clinical manifestations usually develop early in the life and invasive procedures induce remarkable suffering [20]. Experience using continuous desferoxamine infusion demonstrated that local reactions could result in inadequate compliance in many patients, higher morbidity, and increased costs [21]. Distressed by the illness itself and from iron chelation, thalassaemic subjects show frequently maladaptive coping strategies and high levels of anxiety with psychosocial dysfunction [22-27]. A psychosocial support aimed at reducing emotional distress, improving the compliance to chelation therapy, and strengthening the coping strategies for a better integration in daily life, is therefore necessary. Aydinok et al. reported that the frequency of psychopathology is higher in thalassaemic subjects compared to the normal population; this supports the need for thalassaemic patients and their parents of a lifelong psychological support to prevent mental health issues [7].

Our study was designed to verify the effectiveness of CBFT in improving compliance to chelation therapy. Moreover we sought to evaluate the influence of CBFT on the behavioral, emotional, and temperamental profile of thalassaemic children (CBCL, MASC, CDI and EAS), and on the quality of life of their caregiving mothers. 90% of the β-thalassaemic major children included in the study had good compliance, suggesting that CBFT could be effective for this purpose. However the comparison with healthy age-matched children revealed that β-thalassaemic major children have higher scores on somatic complaints, physical symptoms, and separation panic, suggesting an emotional burden. No relevant anomalies in lowered mood were observed in β-thalassemic children. Higher emotionality and reduced sociability were present in thalassaemic children, and increased emotionality and shyness, along with lower sociability, were present in their mothers. An additional goal of the present study was to evaluate the QoL of caregivers. QoL measures could provide helpful information to the family. In caregivers of children with sickle cell disease, another type of chronic hematopathological condition, the quality of life was found significantly lowered on all subscales in comparison with a control group of the same socio-economic status [28]. We have found, similarly, a significant impairment in mothers of β-thalassaemic children in terms of physical health (concerning energy and fatigue, pain and discomfort, sleep and rest subscales), psychological health (bodily image and appearance, feelings, self-esteem, thinking, learning, memory and concentration subscales) and domains assessing overall QoL. The awareness of the illness has been suggested as a possible factor influencing both the compliance with treatment and the quality of life of mothers. In fact, a previous study found that thalassemic children and their parents, who were more conscious of the illness, were also more compliant with the therapy, but also needed more psychological support, being affected by depression, obsession, paranoia, and hostility [7].

Our data agree with these studies, as mothers of our thalassaemic well compliant children elaborated a painful perception of the disease and showed impairment in domains involved in physical health, psychological health, quality of life and general health. Poor QoL of caregiving mothers could be explained by a sense of guilt for having generated a child with a genetically determined disease. CBFT could be considered as an appropriate approach to β-thalassaemic children because it seems to have a positive effect on the compliance with the chelation therapy, thus improving the relation and communication with medical staff; however its role is questionable for the complexity of the presenting symptoms both on children and parents. Mothers often conceal their emotional distress, and their attitude is generally overprotective towards the thalassemic children.

Despite some limitations, our study is provocative and has potential implications for future research. However, it should be viewed in the context of the following considerations. 1. Endemic Countries in the Mediterranean region have achieved 80–100% prevention of newly affected births, by long-established control programs based on avoiding the pregnancies in heterozygotic couples, and this has dramatically reduced the incidence of β-thalassaemia major in Sicily: the small sample size of the present study reflects these prevention strategies 2. Considering that all the patients were psychologically stressed, we had to make the choice of including each of them in the CBFT sessions, to ensure them the adequate support. 3. In order to assess the degree to which the effects of a sustainable psychological support could influence the experience of parenting a child with thalassaemia major, a longitudinal study of changes over the life span of thalassaemic people, to be started before the initiation of the support therapy, becomes necessary.

## Conclusion

The present study suggests that CBFT can be an additional tool to approach the complexity of a chronic disease such as β-thalassaemia major. The choice of CBFT can be can be justified to improve the compliance to chelation therapy; however certain psychological findings, such as somatic complaints, physical symptoms, and separation panic in β-thalassaemia major children are indicative of persistent emotional burden, probably related to the fear for future complications. Involvement of the mothers in the therapeutic setting can improve the relationship with child and medical staff, but we also have to consider that in this case the quality of life of mothers remains somewhat compromised. This kind of therapy could therefore be combined with additional psychological support.

## Competing interests

The authors declare that they have no competing interests.

## Authors' contributions

LM and DM designed the study, performed statistical analysis and drafted the manuscript. LB and FA collected the data. MAR assisted in design of the study. All authors read and approved the final manuscript.
